# Clomiphene citrate versus letrozole with gonadotropins in intrauterine insemination cycles: A randomized trial

**Published:** 2017-01

**Authors:** Leila Pourali, Sedigheh Ayati, Shirin Tavakolizadeh, Hourieh Soleimani, Fatemeh Teimouri Sani

**Affiliations:** 1 *Department of Obstetrics and Gynecology, Faculty of Medicine, Mashhad University of Medical Sciences, Mashhad, Iran. *; 2 *Mashhad University of Medical Sciences, Mashhad, Iran.*

**Keywords:** Ovarian stimulation, Gonadotropin, Letrozole, Clomiphene citrate

## Abstract

**Background:**

Clomiphene citrate is one of the effective drugs for infertility treatment due to oligo-ovulation or anovulation. Intrauterine insemination (IUI) is one of more adherent methods for treatment of infertile cases which is followed by controlled ovarian hyperstimulation (COH).

**Objective:**

the aim of this study was to evaluate Clomiphene citrate versus letrozole with gonadotropins in IUI cycles.

**Materials and Methods:**

In this prospective randomized trial, 180 infertile women who were referred to Milad Hospital were selected. The first group received 5 mg/day letrozole on day 3-7 of menstrual cycle. The second group received 100 mg/day Clomiphene in the same way as letrozole. In both groups, human menopausal gonadotropin was administered every day starting on day between 6-8 of cycle. Ovulation was triggered with urinary Human Chorionic Gonadotropin (5000 IU) when have two follicles of ≥16 mm. IUI was performed 36 hr later.

**Results:**

The number of matured follicles, cycle cancellation, and abortion were the same in both groups. Endometrial thickness was higher at the time of human menopausal gonadotropin administration in letrozole group. Chemical and clinical pregnancy rates were much higher in letrozole group. Ovarian hyperstimulation was significantly higher in clomiphene group.

**Conclusion:**

Letrozole appears to be a good alternative to clomiphene citrate with fewer side effects.

## Introduction

Infertility is commonly defined as the failure of conception after at least twelve months of unprotected intercourse (1). According to a research in Iran, the overall prevalence of infertility was 8% (2). IUI (Intrauterine Insemination) may be recommended as a first-line treatment in young couples with different etiologies of infertility such as male factor infertility, unexplained infertility and ovulatory disorders (3). Clomiphene citrate (CC) is a selective estrogen-receptor modulator (SERM) which is the most commonly prescribed agent to induce ovulation (4). It has been widely used in treatment of infertility since its introduction into clinical practice (5). Clomiphene results in a 60-85% ovulation rate and a 10-20% pregnancy rate per cycle (6). But there are many studies which show that the clomiphene has significant adverse effects on endometrial receptivity, endocervical mucosa, fetus and ovaries (7, 8). Anti-estrogenic effects of CC on the endometrium may lead to poor pregnancy rate and significant rate of early pregnancy loss by the mechanism of estrogen receptor (ER) depletion (9). CC has a long half-life, so it accumulates in the body and has adverse effects as mentioned (4).

Letrozole is a third-generation aromatase inhibitor which has been successfully used for ovulation induction in patients with polycystic ovary syndrome (PCOS) (11). Mitwally *et al* had reported acceptable pregnancy outcomes and lower rate of multiple gestation in letrozole group for ovarian stimulation (12). Letrozole does not deplete estrogen receptor (ER) in target tissues, so it has no persistent anti-estrogenic effect. It typically results in mono-ovulation and it may have no adverse effects on endometrium and cervical mucosa. It has a short half-life (45 hr), so it would be eliminated from the body rapidly (13). Although clomiphene is the standard drug for ovulation stimulation, clomiphene-resistance has been discovered in 15-20% of the patients (11).

The aim of this prospective randomized clinical trial was to compare the efficacy of Letrozole +HMG with Clomiphene +HMG in a group of patients with unexplained infertility that had failed to conceive after previous treatment by CC alone.

## Materials and methods

This randomized double-blind clinical trial was performed at Milad infertility clinic, Mashhad, Iran, between April 2010 and March 2011. For sample size calculation with regard of α=0.05 and β=0.2, and data from a previous study which showed that the clinical pregnancy rate was 14 % in CC group and 32% in letrozole group, according to the formula, after assuming a 5% dropout rate, to reach the minimal statistically-acceptable figure, a minimal sample size of 90 cases was calculated in each group (13). 

**Figure 1.**
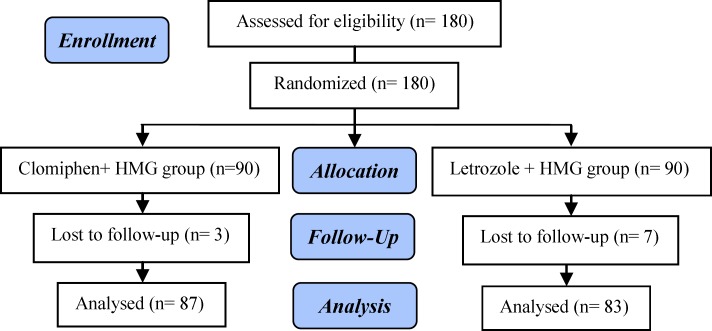
Consort flowchart of the study design

So, we enrolled 180 infertile women who were eligible for superovulation and IUI for the first time. Inclusion criteria were unexplained infertility and resistance to three cycles of clomiphene therapy who were candidate for IUI (Figure 1). Exclusion criteria were women with PCOS, thyroid dysfunction, hyperprolactinemia, endometriosis, ovarian hyperstimulation (more than 15 follicles in each ovary) and age more than 38 yr. Hysterosalpingography was performed for all participants to confirm tubal patency. Semen parameters were analyzed by the world health organization (2010) criteria (13). 

The patients were randomized in two groups: Clomiphene group (Clomiphene +HMG) and Letrozole group (letrozole +HMG). Randomization was done by using numbers in closed envelops. The patients in the clomiphene group received CC 50 mg twice a day (BID) for 5 days starting from day 3 of menstrual cycle. In letrozole group, letrozole (Femara, Novartis, Quebec, Canada) 2.5 mg BID was given for 5 days from day 3 of the menstrual cycle. 

In addition, all the patients received a daily intramuscular (IM) human menopausal gonadotropin (HMG, Pergonal, Serono, Switzerland) injection. The dosage was 75 IU starting on day 6 of menstrual cycle until hCG administration. The gynecologists, radiologists and participants were unaware of study group allocation. Drugs and treatment protocol was given by the medical consultant team to the participants. Transvaginal ultrasonography was done in the days 3, 9, 12 of the cycle and then every three days, until follicle size reached more than 16 mm in size. 

If more than 15 follicles were seen in each ovary that is considered as ovarian hyperstimulation, so the cycle was cancelled and the participant was excluded from the study. When mature leading follicle(s) reached >16 mm in diameter, urinary hCG (Profasi, Serono, Italy) in a dose of 5,000 IU was given and IUI was performed 36 hr later. two weeks after the performance of IUI, if the participant was not menstruated, chemical pregnancy would be defined by the measurement of βHCG level. 

Transvaginal ultrasonography (TVS) was done four weeks after positive pregnancy test to confirm the presence of gestational sac with fetal pole and fetal heart pulsation, so the clinical pregnancy was identified. Finally, the therapeutic costs were calculated and compared between groups.

**Ethical Consideration**

After approval by the medical ethics committee of Mashhad university of Medical Science; all the participants were aware of the purpose and procedures of the study and informed consent was obtained.

**Statistical analysis**

Statistical Package for Social Sciences, version 16.0, SPSS Inc, Chicago, Illinois, USA (SPSS 16) was used for statistical analysis and *t* test, Fisher-exact test and chi-square test were used as appropriate. P<0.05 was considered statistically significant.

## Results

180 participants were included in the analysis, 87 (51.2%) in clomiphene group and 83 (48.8%) in the letrozole group. ten women were excluded from the study due to lack of following their treatment. There were no significant differences between women in both groups in terms of age, body mass index (BMI), duration and types of infertility (Table I). 

The mean endometrial thickness on the day of hCG administration was significantly higher in letrozole group (8.99±0.65 vs. 8.46±0.69 mm respectively ,p=0.001). The clinical pregnancy rate was significantly higher in letrozole group which is defined by fetal heart detection in ultrasonography (26.51 vs. 12.46% respectively, p=0.001). No twin or ectopic pregnancy occurred in this study.

Four patients (5.7%) in letrozole group and five patient (4.8%) in CC group experienced abortion; there was no significant difference between two groups (p=0.80). Ovarian hyper stimulation, determined by the number of follicles more than 15, was significantly higher in clomiphene group (5.7 vs. 0%, respectively, p=0.03). 

The mean number of mature follicles (≥16 mm) was not significantly different between clomiphene and letrozole group (2.17±0.13 vs. 2.28±0.1%, respectively, p=0.74). Cancelled cycles occurred due to ovarian hyperstimulation syndrome was significantly higher in clomiphene group compared to letrozole group (5.7 vs. 0 %, respectively, p=0.027). There was no significant difference between clomiphene and letrozole groups in terms of formation of at least two follicles with size of ≥16 mm (8 vs. 6%, respectively, p=0.607). Therapeutic costs including all drugs, ultrasonography, visits, and IUI costs were approximately the same between two groups (p=0.62).

**Table I:** Comparison of patients treated with Clomiphene and letrozole groups

**Variable**		**Clomiphene +HMG group**	**Letrozole+ HMG group**	**p-value**
Age (yr)		28.5 ± 1.7	28.6 ± 1.8	0.850*
Duration of infertility (yr)		2.5 ± 1.14	2.5 ± 1.19	0.902*
BMI (Kg/m²)		24.86 ± 2.6	24.6 ± 2.7	0.611*
Endometrial thickness before treatment (mm)		3.7 ± 0.86	3.8 ± 0.85	0.553*
Endometrial thickness during HCG injection (mm)		8.46 ± 0.69	8.99 ± 0.65	0.001*
Rate of pregnancy by positive βHCG		16 (18.4%)	26 (31.3%)	0.059**
Rate of pregnancy by fetal heart detection by ultrasonography		11 (12.64%)	22 (26.51%)	0.022**
Rate of abortion (The recent pregnancy)		5 (5.7%)	4 (4.8%)	0.80**
Ovarian hyperstimulation (follicles more than 15)		5 (5.7%)	0	0.03***
Therapeutic cost (Rials)				
	≤ 5,000,000	72 *(82.8%)	71 (85.5%)	0.62**
	10,000,000 ≥ Cost ˃ 5,000,000	15 (17.2%)	12 (14.5%)	0.62**
No. of follicles ≥ 16 mm		2.17 ± 0.13	2.28 ± 0.1	0.74**
Rate of cancelled cycles				
	Due to OHSS	5 (5.7%)	0	0.027***
	Due to non-formation of at least 2 follicles ≥ 16 mm	7 (8%)	5 (6%)	0.607***

* Student's *t*-test

** Chi-square test

*** Fisher-exact test

## Discussion

The results from this study indicate that endometrial thickness was significantly higher in letrozole group; similar to some other studies (13,15,16); but in some studies, there was no significant difference in terms of endometrial thickness between two groups (14-18). This difference could be due to ovulatory dysfunction in their participants which may play a role in endometrial thickness. In the current study, there was no significant difference in the number of follicles which is more than 16 mm in size, just like as the study of Zadehmodares *et al*, but in the study of Badawy *et al*, there were more follicles ≥16 mm in clomiphene group that may be due to their larger sample size, also their study was done on patients with polycystic ovarian disease (PCOD) which were more susceptible to have higher number of follicles (17, 19). 

In this study, there was a significant difference in clinical pregnancy rate which was much higher in letrozole group, similar to some studies, but in some other studies, there was no significant difference in pregnancy rate like as Zadehmodares *et al* and Akbari *et al* studies that may be due to their smaller sample size (13, 15, 16, 19, 20). The higher pregnancy rate in letrozole group can be explained by significant increase in endometrial receptivity as assessed by endometrial thickness. The lower pregnancy rate in CC may be due to antiestrogenic effects of CC on the endometrium and cervical mucusa. In the present study, there was no significant difference between two groups in terms of abortion. The systematic review which was done on this field also showed no difference in the abortion rate (21).

This study showed significantly more OHSS in clomiphene group, this important complication does not have much incidence rate in some other studies, even a systematic review in this field did not show this difference (9, 10, 21). This is a very important problem because this is a life-threatening complication and also it is the most important cause of cancelling the COH cycles in which in the current study, there was significant difference in terms of cycle cancelling in cc group; so may be the use of lower dose of cc must be considered in our protocols. 

The therapeutic cost in both protocols was the same; we did not find any study in this field. Some of the strength of current study include: double blind randomization design and evaluation of treatment costs in two groups. 

**Limitation**

The limitations of this study were: 1. Short period of follow-up after pregnancy which cannot detect pregnancy outcome (after 20 weeks) and also the teratogenic effects of these drugs, 2. More than one sonologist had done the serial ultrasonography for evaluation of endometrial thickness and follicular growth, so it could be a weakness of this study.

## Conclusion

Letrozole has beneficial effect on endometrium which may improve the pregnancy rate in women with unexplained infertility.

## Note

***This article extracted from M.D. thesis. (Shirin Tavakolizadeh)***

***Registration ID in IRCT:*** ***IRCT2015062422900N1***
